# Cell and molecular mechanisms of keratinocyte function stimulated by insulin during wound healing

**DOI:** 10.1186/1471-2121-10-1

**Published:** 2009-01-12

**Authors:** Yan Liu, Melissa Petreaca, Min Yao, Manuela Martins-Green

**Affiliations:** 1 Burn Department, Ruijin hospital, Shanghai JiaoTong University Medical School, Shanghai, PR China; 2 Department of Cell Biology and Neuroscience, University of California, Riverside, CA, USA

## Abstract

**Background:**

Regenerative wound repair is a goal of modern medicine. This is important not only for the local repair but also for its beneficial effect to systemic physiological processes. When wounds become chronic, individuals are susceptible to generalized inflammatory cascades that can affect many organs and even lead to death. Skin is the most commonly injured tissue, and its proper repair is important for reestablishment of its barrier function.

**Results:**

We show here that insulin, when topically applied to skin excision wounds, accelerates re-epithelialization and stimulates "maturation" of the healing tissue. These effects are dependent on the insulin receptor but independent of EGF/EGF-R; PI3K-Akt-Rac1 signaling pathways are critically involved, and healing is α3 and LN332-dependent.

**Conclusion:**

Insulin has great potential for the treatments of chronic wounds in which re-epthelialization is impaired. Understanding of the pathways induced by insulin is important for the development of analog molecules that function strictly in healing. Because of its long history of safe use in humans for decades, this protein may prove to be a powerful therapy without major adverse effects.

## Background

Wound healing is a complex process that involves sequential phases that overlap in time and space, interact, and affect each other dynamically both at the gene and protein levels. In addition, crosstalk between cells and the surrounding microenvironment contributes to the processes of clot formation, inflammation, granulation tissue development, and remodeling. Many different lines of experimental evidence have shown that the basic cellular and molecular mechanisms that result in these events involve cell adhesion/de-adhesion, migration, proliferation, differentiation, and apoptosis. One important process initiated during the early stages of healing is re-epithelialization; it involves the proliferation, migration, and differentiation of keratinocytes from the wound margins [1,2]. Appropriate re-epithelialization requires not only the development of a continuous epidermal layer but also full epidermal differentiation and the formation of junctions between the epidermis and dermis. Because the epidermis provides a barrier against infection and maintains homeostasis, improving re-epithelialization, particularly in impaired healing situations, has attracted a great deal of attention. One molecule with the potential to enhance these processes is insulin, a hormone known to maintain the growth and development of different cell types. It can affect the proliferation, migration [3], and ECM secretion by keratinocytes, endothelial cells, and fibroblasts [4].

The use of insulin for non-diabetic purposes was popular in the early part of the 20th century [5,6], was "forgotten" during the 40's and 50's, and it became again reinvigorated during the latter half of the century. For example, daily injections of insulin were used to improve bone healing in rats [7,8], incision wounds of the skin [9,10], healing in the distal limb of horses [11], and in cutaneous ulcerations in diabetic and non-diabetic mice [12]. Insulin was also used in the 60's to treat diabetic wounds in humans [13,14], and more recently, insulin spray has been successfully used to treat patients with diabetic ulcers. Furthermore, this hormone has been used to treat burns in humans [15], rats [16], and rabbits [17] with good success. In addition to the studies *in vivo*, experiments with cultured cells have shown that insulin increases the rate of growth of fibroblasts, cells that are critically involved in the development of the granulation tissue [18], suggesting that insulin can function as a growth hormone [19]. Nevertheless, despite the strong evidence that insulin stimulates healing and thereby decreases the time of wound closure, the underlying mechanisms of insulin-induced improved healing are far from understood.

Here we show that local application of insulin to excision wounds stimulates keratinocyte migration and differentiation, and that this is dependent on activation of the PI3K-Akt pathway, followed by activation of Rac1, and that the integrin α3 and the ECM molecule laminin 332 (LN332), are critical. We also show that insulin stimulates a regenerative process in the wound tissue. Therefore, insulin may prove to be useful in the treatment of chronic wounds, dental/gum healing problems, and burns. These are important findings because issues of impaired healing and of lack of tissue regeneration have implications for numerous health- and financially-related problems in this country and elsewhere. The understanding of key elements of the signaling pathways induced by insulin during stimulation of healing can lead to the development of analogs that will function strictly in the healing process.

## Results

### Topical application of insulin accelerates and improves the quality of healing

**Figure 1:**
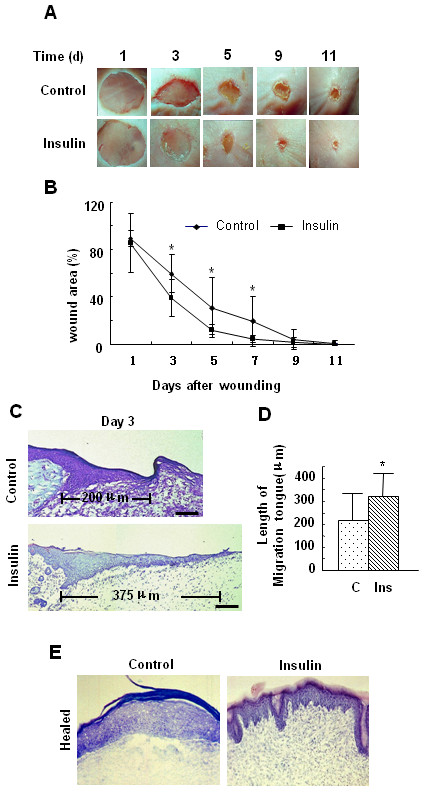
**Insulin accelerates wound healing and improves the quality of re-epithelialization**. Excision wounds were performed in C57BL/6J mice, and the healing process was monitored at different time points. **(A) **Representative images of wounds which were treated with vehicle (30 μl saline solution) or 0.03 u insulin/30 μl saline solution every two days. **(B) **Wound area was quantified every two days and expressed as the percentage of the original wound area (n = 9; Statistics are shown as comparisons between the treatment and control. *P < 0.05). Insulin significantly decreased wound area. **(C, D) **The length of the migrating tongues was determined by measuring the distance from the margin of the wound to the tip of the migrating keratinocytes at 40× magnification, in which one unit equals 25 μm. **(C) **Representative hematoxylin and eosin-stained sections showing longer migrating tongue in insulin treated wounds. Scale bars indicate 50 μm. **(D) **Data are shown as the mean value +/- SD. Statistics are shown as comparisons between the treatment and control (n = 9). *P < 0.05. **(E) **Representative hematoxylin and eosin-stained sections showing increased number of epidermal reticular ridges and dermal papilla in insulin treated healed wounds.

To study the effects of insulin on wound healing, 7 mm diameter excision wounds were performed on the back of C57BL/6J mice, and locally treated with 0.03 U of insulin. This dose of insulin was chosen because it significantly stimulated healing (Fig. 1A) without affecting blood glucose levels (unpublished data). We analyzed the wound area throughout the healing process to monitor the time-dependent effects of insulin on healing, and found that in wounds treated with insulin the wound area was significantly decreased at several time points (Fig. 1B), as was the time to closure (control 10.25 ± 1.26 d, insulin 8.9 ± 0.32 d, P < 0.01). We found that insulin significantly decreased wound area by day 3 after injury. In order to elucidate the effects of insulin during this early stage of healing, we took skin samples from control and insulin-treated wounds, and compared the histological characteristics of these two wounds. At day 3, we found that in insulin-treated wounds the keratinocyte tongue was much longer than that in the control wounds, suggesting that insulin stimulates keratinocyte migration (Fig. 1C). When we measured the extent of migration of the keratinocytes by determining the length of migration of the tongue from the margin of the wound to the tip of the migrating keratinocytes, we found that there was a significant increase in migration distance of the keratinocytes in insulin-treated wounds (Fig. 1D). Furthermore, we also found that following wound closure, the epidermis of insulin-treated wounds is better defined and is characterized by an increased number of epidermal reticular ridges and dermal papilla that are not evident in the control (Fig. 1E).

### Insulin stimulates keratinocyte migration in a time- and dose-dependent manner

**Figure 2:**
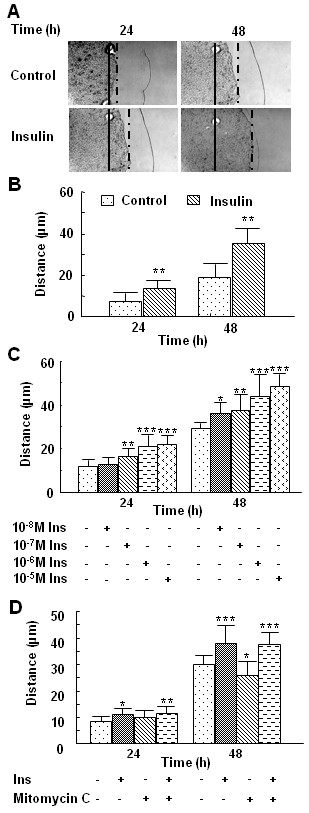
**Insulin stimulation of keratinocyte migration is time- and dose-dependent**. Cells were assayed for migration using the cloning ring migration assay. **(A, B) **Cells were treated with 10^-6 ^M insulin at multiple time points, as indicated. Insulin significantly increased keratinocyte migration. **(C) **Cells were then treated with 10^-8 ^M, 10^-7 ^M, 10^-6 ^M or 10^-5 ^M insulin, and migration distance measured 24 and 48 h after insulin treatment. The effect of insulin on keratinocyte migration was dose-dependent. **(D) **Cells were pre-treated with 5 μg/ml mitomycin C for 3 h followed by 10^-7 ^M insulin, and migration distance measured 24 and 48 h after insulin treatment. The effect of insulin on keratinocyte migration was not abolished by mitomycin C. Each treatment group was performed in triplicate. Data are shown as the mean value +/- SD. Statistics are shown as comparisons between the treatment and control. *P < 0.05, **P < 0.01. ***P < 0.001.

Histological observation of wounds treated with insulin suggests that this protein stimulates keratinocyte migration. Although the effects of insulin on keratinocyte proliferation are well established [20], its effect on migration of these cells is not clear. To study the latter process, we used HaCaT keratinocytes in culture. Cells were plated in cloning rings and allowed to attach; the rings were removed after marking their positions and the cells were then treated with insulin. Migration distances from the initial edge of the cells to the new edge of the cells were measured at 24, 48, and 72 hr. At each time point, keratinocytes treated with insulin showed increased migration over the control (Fig. 2A, B). To determine whether this effect is dose-dependent, we performed the migration assays with different doses of insulin. A concentration as low as 10^-8 ^M insulin was able to increase keratinocyte migration, which was highly significant after 48 hr of treatment. However, concentrations ranging from 10^-7 ^M to 10^-5 ^M significantly enhanced keratinocyte migration by 24 h (Fig. 2C). To eliminate the possibility that this migration is dependent on proliferation, we treated the cells with mitomycin C, a potent DNA crosslinker and hence inhibitor of cell proliferation, in the presence or absence of insulin. Cells were pre-treated with 5 μg/ml of mitomycin C for 3 hrs and then exposed to 10^-7 ^M insulin for 24 and 48 hrs. Although we observed that proliferation was halted, insulin-induced migration was not (Fig. 2D), strongly suggesting that the two processes are independently regulated by this hormone.

### Insulin stimulates keratinocyte migration in an insulin receptor-dependent manner but in an EGF-independent manner

**Figure 3:**
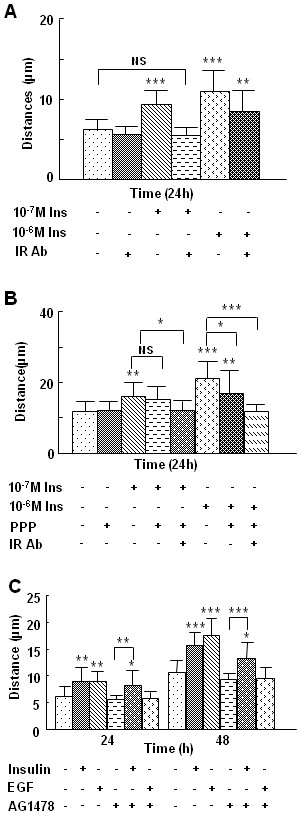
**Insulin-induced keratinocyte migration is insulin receptor-dependent and EGFR-independent**. HaCaT Cells were plated in the cloning ring migration assay as described. **(A) **Cells were pre-treated with 1.5 μg of neutralizing insulin receptor Ab 29B4 for 1 h, then treated with 10^-7 ^M or 10^-6 ^M insulin for 24 h. Neutralizing insulin receptor Ab inhibited 10^-7 ^M but not 10^-6 ^M insulin-induced cell migration, showing that the effect of 10^-7 ^M insulin on keratinocytes is mediated primarily through the insulin receptor. **(B) **Cells were pre-treated with either 50 nM IGF-1 receptor inhibitor picropodophyllin, 1.5 μg neutralizing insulin receptor Ab 29B4, or insulin receptor Ab 29B4 plus picropodophyllin for 1 h, then treated with 10^-7 ^M or 10^-6 ^M insulin or left untreated for 24 h. The effects of a higher dose of insulin but not a lower dose are mediated by both the insulin receptor and the IGF-1 receptor. **(C) **Cells were pre-treated with 3 μM of the EGF-R inhibitor AG1478 for 1 h, followed by treatment with 10^-7 ^M insulin; migration distance was measured at 24 and 48 h after treatment. Insulin-induced migration was not prevented by the EGF-R inhibitor. Each treatment group was performed in triplicate. Data is shown as the mean value +/- SD. Statistics are shown as comparisons between the treatment and control, unless otherwise indicated. *P < 0.05, **P < 0.01. ***P < 0.001.

It has been shown that insulin activates both its own receptor and the IGF-1 receptor, albeit with different affinities [21]. These transmembrane proteins are both tyrosine kinases, share 60% homology, and activate a number of insulin receptor substrates which then initiate signals that lead to gene expression. These genes are involved in many of the different effects of insulin on cells and ECM molecules, as well as their receptors such as integrins, which provide critical signals to guide cell movement. To determine whether insulin-induced keratinocyte migration is dependent on one or both receptors, each group of cells was pre-treated with either the neutralizing insulin receptor Ab, 29B4, or the IGF-1 receptor tyrosine kinase inhibitor, picropodophyllin and then treated with 10^-7 ^M or 10^-6 ^M insulin. Pre-treatment of keratinocytes with the insulin receptor Ab followed by 10^-7 ^M insulin treatment completely abolished insulin-induced migration, suggesting that at this concentration these effects are primarily mediated by the insulin receptor itself (Fig. 3A). When cells were treated with the insulin receptor Ab and 10^-6 ^M insulin, the Ab only partially blocked insulin-induced migration (Fig. 3A), suggesting that this concentration of insulin may induce migration through both the insulin and IGF receptors. To confirm these results, we pre-incubated the cells with picropodophyllin for 1 hr and then treated them with insulin. At 10^-7 ^M, insulin-induced keratinocyte migration was not affected, but the inhibitor did decrease cell migration induced by 10^-6 ^M insulin (Fig. 3B). Moreover, the keratinocyte migration resulting from 10^-6 ^M insulin was abrogated when inhibiting both insulin and IGF receptors using the 29B4 Ab and picropodophyllin (Fig. 3B). Taken together, these data show that the effect of high concentration (10^-6 ^M) of insulin on keratinocyte migration is mediated by both insulin and IGF-1 receptors, whereas the effect of lower concentrations of insulin (10^-7 ^M) is primarily mediated by the insulin receptor. To study the effects of insulin that are mediated only through the insulin receptor and its associated downstream signaling pathways, 10^-7 ^M insulin was chosen for the subsequent studies except when otherwise indicated.

Our previous studies showed that insulin stimulates EGF expression in wound marginal keratinocytes of deep partial thickness scald wounds in rats [16]. In order to exclude the potential autocrine effects of EGF secretion on insulin-induced keratinocyte migration, we treated the keratinocytes with AG1478, a selective inhibitor of EGF-R kinase, prior to treatment with insulin and measured migration distances at 24 and 48 h. This EGF-R inhibitor did not inhibit the effects of insulin on keratinocyte migration (Fig. 3C), suggesting that this process does not require EGF or its receptor.

### PI-3K and Akt mediate insulin-induced keratinocyte migration

**Figure 4:**
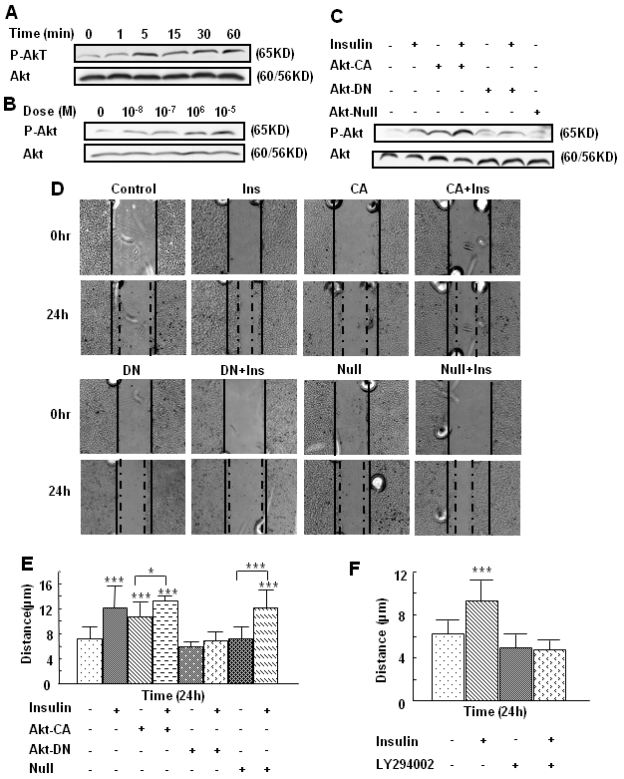
**PI-3K and Akt mediate insulin-induced keratinocyte migration**. **(A) **HaCaT cells were either left untreated or treated with 10^-7 ^M insulin for the indicated time points, followed by immunoblot analysis of the cell lysates using an Ab that specifically recognizes phosphorylated Akt at Ser 473. This blot was reprobed with an Ab to Akt to ensure equal loading. Insulin increased Akt phosphorylation over time, with peak phosphorylation seen at 5 min. **(B) **HaCaT cells were either left untreated or treated with 10^-8 ^M to10^-5 ^M insulin for 5 min, and then analyzed as in (A). Insulin stimulated phosphorylation of Akt in a dose-dependent manner. **(C) **HaCaT cells grown to 60–70% confluence were infected with adenovirus expressing constitutively active Akt (Akt-CA), dominant-negative Akt (Akt-DN) or the null adenovirus (Null) by incubating cells with the adenovirus for 5 h. Forty-eight hours after infection, cells were treated with 10^-7 ^M insulin for 3 min or left untreated. Phosphorylation of Akt and total Akt were detected by immunoblot. The Akt-DN inhibited insulin-induced Akt phosphorylation. **(D, E) **HaCaT Cells grown to 50–60% confluence were infected with adenovirus expressing Akt-CA, Akt-DN, or null adenovirus, as mentioned above. Forty-eight hours after infection, scratch wounds were made, and cells were treated with10^-7 ^M insulin; migration distances were measured after 24 h of treatment. Again expression of the Akt-DN inhibited insulin-induced migration. **(F) **HaCaT cells were incubated with 50 μM of the PI3K inhibitor LY294002, 10^-7 ^M insulin, or LY294002 for 1 h, followed by treatment with 10^-7 ^M insulin. The migration distances were measured 24 h after treatment. Insulin induced keratinocyte migration was abolished by pre-treatment with the PI3K inhibitor LY294002. Results are representative of at least three independent experiments. *P < 0.05 *vs *control; ***P < 0.001 *vs *control.

In order to determine the signal transduction pathways in insulin-induced keratinocyte migration, we examined Akt phosphorylation/activation by immunoblot analysis [22,23], and found that the levels of phosphorylation of this signal transduction mediator increased after 5 min of insulin treatment, and remained elevated for at least 60 min (Fig. 4A). This effect was also dose-dependent (Fig. 4B). To determine whether Akt phosphorylation/activation was involved in insulin-induced keratinocyte migration, we infected keratinocytes with recombinant adenovirus expressing the constitutively active mutant of Akt (Akt-CA) or the dominant-negative mutant of Akt (Akt-DN). Higher levels of p-Akt were found in the keratinocytes expressing Akt-CA even without insulin treatment when compared to cells expressing the Akt-DN (Fig. 4C). To determine whether Akt phosphorylation is important in keratinocyte migration, we used the scratch wound migration assay. This assay was used to avoid trypsinizing and re-plating the cells because the viral infected cells have decreased survival. We compared keratinocyte migration distances with or without insulin treatment, in cells expressing the Akt mutants (Fig. 4D, E). Uninfected cells and cells infected with the null vector and then treated with insulin as well as cells infected with the Akt-CA, with or without insulin treatment, displayed the longest migration distance. In contrast, cells infected with Akt-DN exhibited a significant decrease in migration, even with insulin treatment, compared with all other groups, illustrating the requirement of Akt for insulin-induced keratinocyte migration.

PI3K is often involved in AKT phosphorylation. Therefore, to determine whether insulin stimulation of keratinocyte migration is dependent on PI3K activity, we performed the migration assays in the presence of LY294002, an inhibitor of PI3K. This treatment completely blocked keratinocyte migration stimulated by insulin (Fig. 4F), showing the importance of PI3K in this process. The dose of LY294002 we used does not inhibit S6 kinase, which is the effector of mTOR, nor does it affect MAP kinase, PKC, or PI4K [24].

### Insulin stimulates translocation of Rac1, but not RhoA, to the plasma membrane; this process requires PI3K-Akt activation and is involved in insulin-induced keratinocyte migration and wound healing

**Figure 5:**
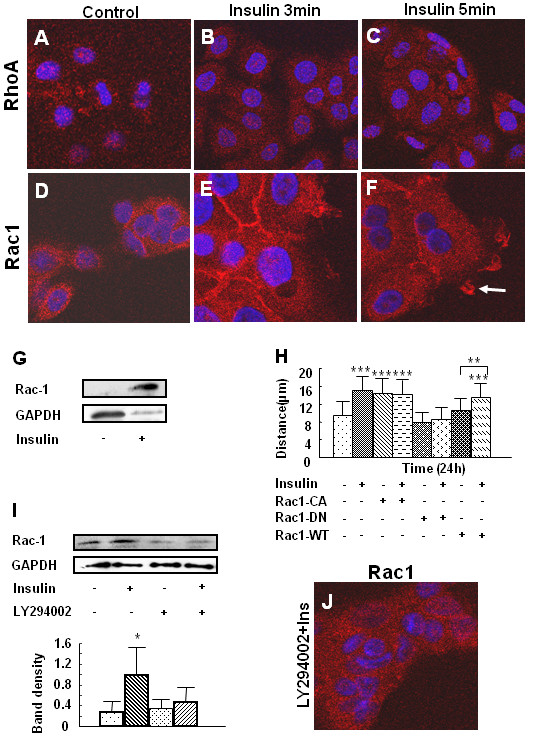
**Insulin stimulates Rac1 activation through PI3K-Akt in insulin-induced keratinocyte migration**. **(A-H)**. Cells were seeded in 8 well chamber slides for at least 24 h before the various treatments and then fixed and stained for either RhoA or Rac1 to visualize these molecules in the cell. **(A-F) **Cultures were either left untreated or treated with 10^-7 ^M insulin for 3 or 5 min. **(A-C) **Staining for RhoA: **(A)**, control, **(B)**, insulin treatment 3 min **(C)**, insulin treatment 5 min. Insulin does not stimulate translocation of RhoA to the cell membrane. **(D-F) **Staining for Rac1: **(D)**, control. **(E, F)**, insulin treatment at 3 and 5 min. Arrowhead shows membrane ruffles. Insulin induces Rac1 membrane translocation as well as membrane ruffling in migrating cells. **(G) **Cells were treated with insulin for 5 min and a Rac1 pull-down assay performed. Insulin induced Rac1 activation. **(H) **Cells were transfected using lipofectin with plasmids expressing the constitutively active form of Rac1 (V12, Rac1-CA), the dominant-negative mutant of Rac1 (N17, Rac1-DN) and the wild type Rac1 (Rac1-WT). Forty-eight hours after transfection, scratch wounds were made, and cells were treated with 10^-7 ^M insulin. Cell migration was monitored for 24 h after insulin. Insulin-induced keratinocyte migration was eliminated by Rac1-DN. Each treatment group was performed in triplicate. Data is shown as the mean value +/- SD. ***P < 0.001 vs control treatment. **(I, J) **Cells were pre-treated with 50 μM LY294002 for 1 h followed by 10^-7 ^M insulin treatment for 5 min. **(I) **Rac1 pull-down assay was then performed as mentioned above. Results are representative of three independent experiments. Levels of the active form of Rac1 were quantified by determining the ratio of the integrated density of the GTP binding form of Rac1 to GAPDH, the loading control, using ImageJ software. Data are shown as the mean value +/- SD. *P < 0.05 vs control treatment. Both Rac1 activation (I) and translocation to the plasma membrane (J) were inhibited by LY294002 pre-treatment.

Small GTPases of the RhoA family play important roles in cell motility. Therefore, we tested the possibility that the PI3K-Akt pathway stimulates RhoA activation during insulin-induced keratinocyte migration. Using immunolabeling, we show that there was no significant difference in RhoA distribution shortly after insulin treatment (Fig. 5A–C) nor did a change occur at least for 4 h. However, 3 min after insulin treatment, Rac1, another member of the RhoA family of GTPases, translocated from the cytosol to the plasma membrane, indicating its activation (Fig. 5E). This redistribution effect was also seen after 5 min of insulin treatment (Fig. 5F). In addition, plasma membrane ruffling was observed at the leading edge of migrating keratinocytes, with Rac-1 being present in the membrane of ruffles (Fig. 5F; arrow). To determine whether this translocation is consistent with the activation of Rac1, we performed Rac1 pull down assays, which can specifically pull down the active form of Rac, and found elevated levels of active Rac1 after insulin treatment (Fig. 5G). In order to confirm that Rac1 activation is important in insulin-induced keratinocyte migration, we transfected keratinocytes with plasmids containing mutant forms of Rac1, and then observed the effects of these mutants on insulin-stimulated cell migration. Keratinocytes were transfected with either the constitutively active form of Rac1 (V12, Rac1-CA), dominant-negative mutant Rac1 (N17, Rac1-DN), or wild type Rac1 (Rac1-WT). 24 h after transfection, scratch wounds were made in the cell cultures, and cell migration distances were measured in non-transfected and transfected cells. After treatment for 24 h, insulin stimulated migration in non-transfected cells, as well as in cells transfected with Rac1-CA or Rac1-WT. Cells transfected with Rac1-CA showed increased migration, even without insulin treatment. However, insulin-induced migration was eliminated in cells transfected with Rac1-DN (Fig. 5H). To determine whether the PI3K-Akt pathway is required for insulin-induced Rac1 activation/translocation, LY294002 was used to pre-treat the cells before insulin treatment. Insulin-induced Rac1 activation (Fig. 5I) and translocation (Fig. 5J) was inhibited by this PI3K inhibitor.

### Insulin stimulates integrin a3 and LN332 production, which contributes to insulin-induced keratinocyte migration and wound healing

**Figure 6:**
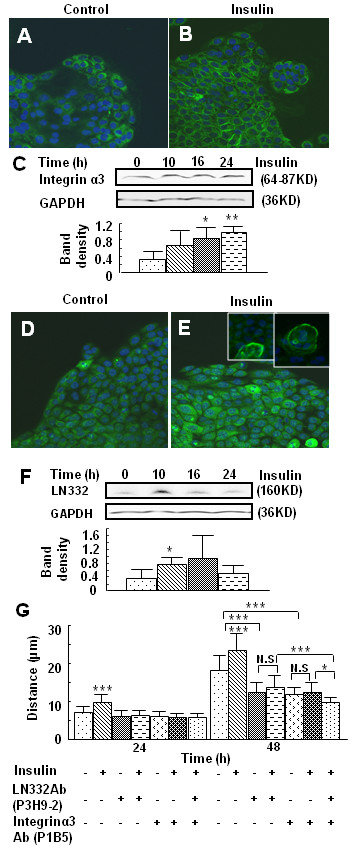
**Insulin-induced integrin α3 and LN332 production contributes to keratinocyte migration**. **(A, B) **Cells seeded in chamber slides were either left untreated or treated with 10^-7 ^M insulin for 24 h, followed by immunolabeling with the anti-integrin α3 Ab and FITC-conjugated secondary Ab. **(A)**, control **(B)**, insulin treatment 24 h. **(C) **Non-reducing western blot analysis of integrin α3 expression shows stimulation of this integrin by insulin in keratinocytes. Results are representative of three independent experiments. Levels of integrin α3 were quantified by determining the ratio of the integrated density of integrin α3 to GAPDH loading control using ImageJ software. Data are shown as the mean value +/- SD. *P < 0.05, **P < 0.01 vs control treatment. **(D, E) **Cells seeded in chamber slides were either left untreated **(D)**, or treated with 10^-7 ^M insulin for 24 h **(E)**, followed by immunolabeling with the anti-LN332 Ab. Small boxes show higher magnification for more detailed distribution of the staining. **(F) **Non-reducing immunoblot analysis of LN332 levels. Insulin stimulates secretion of LN332 by keratinocytes. Results are representative of three independent experiments. Levels of LN332 were quantified by determining the ratio of the integrated density LN332 to GAPDH loading control using ImageJ software. Data are shown as the mean value +/- SD. *P < 0.05 vs control treatment. **(G) **Cells were wounded by scratching and then pre-treated with 25 μg/ml of function-inhibiting Abs directed against LN332 (P3H9-2) and/or integrin α3 (P1B5) for 1 h, then treated with 10^-7 ^M insulin. Migration distances were measured after 24 and 48 h of treatment. *P < 0.05 *vs *control or as indicated. Insulin induced keratinocyte migration was eliminated by both integrin α3 and LN332 function-inhibiting Abs. ***P < 0.001 *vs *control or as indicated. Each treatment group was performed in triplicate.

It is well known that the Rac1 GTPase is critical in cytoskeleton re-organization, that the cytoskeleton interacts with integrins on the cell surface, and that these integrins interact with ECM molecules. It is also known that the integrin α3β1 and LN332, a basement membrane (BM) component, are important in both keratinocyte migration and BM development. Therefore, we investigated the possibility that insulin modulates LN332 and integrin α3β1 expression *in vitro *and *in vivo*, and that these proteins are involved in insulin-induced wound healing. Keratinocytes were seeded in cloning rings in order to observe the integrin α3 and LN332 at the migration edge. Immunolabeling for integrin α3 showed higher levels of this protein on the cell membrane after insulin treatment (Fig. 6A, B). The increased integrin α3 levels were confirmed by immunoblot analysis (Fig. 6C).

Using similar methodology, LN332 was also found to be elevated after insulin treatment, particularly at the migrating edge, with deposition of LN332 along the cell membrane in some cells (Fig. 6D, E and inserts). Immunoblot analysis showed an increase in the LN332 protein after insulin treatment (Fig. 6F). To determine whether these observations translate into changes in the migratory behavior of keratinocytes, we observed the effect of function-inhibiting Abs to integrin α3 and LN332 on insulin-induced keratinocyte migration (Fig. 6G). Cell migration was inhibited when integrin α3 or LN332 were blocked with these Abs, while basal migration remained virtually unaffected. The effects of these blocking antibodies were more obvious after 48 h of insulin treatment; at this time point, functional blocking of α3 and LN332 affected both basal and insulin-induced keratinocyte migration. Moreover, additional inhibition was observed when insulin treatment was accompanied by treatment with both integrin α3 and LN332 function-inhibiting Abs (Fig. 6G).

**Figure 7:**
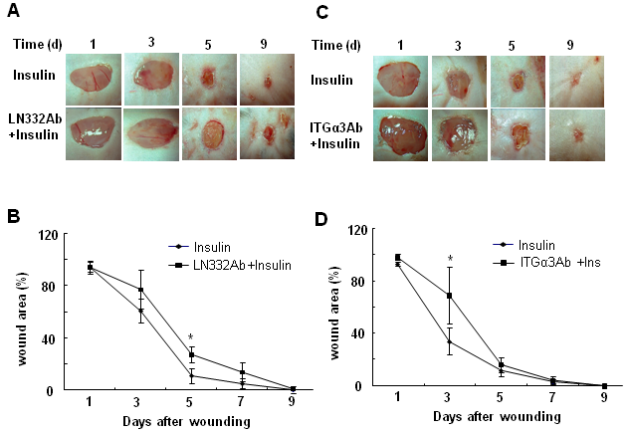
**Integrin α3 and LN332 contribute to insulin-induced accelerating healing**. **(A) **Representative images of excision wounds performed as in Figure 1. After wounding, some wounds were treated with 5 μl LN332 Ab (P3H9-2, 1 mg/ml) for 5 min, followed by 0.03 U insulin/30 μl PBS; other wounds were treated with 0.03 U insulin alone. Treatment was administered every two days until all wounds were closed. **(B) **Wound areas were quantified every two days and expressed as percentage of the area of the control (n = 3). Statistics are shown as comparisons between the LN332 Ab + insulin and insulin treatment. *P < 0.05. **(C) **Excision wounds were performed as above, except that wounds were treated with 5 μl of the anti-integrin α3 Ab (P1B5, 1 mg/ml) rather than the LN332 Ab. Function-inhibiting anti-integrin α3 Ab inhibited insulin-induced wound healing at early stages. **(D) **Wound areas were quantified as in (B). Statistics are shown as comparisons between the anti-integrin α3 Ab + insulin and insulin treatment. *P < 0.05. (n = 3).

**Figure 8:**
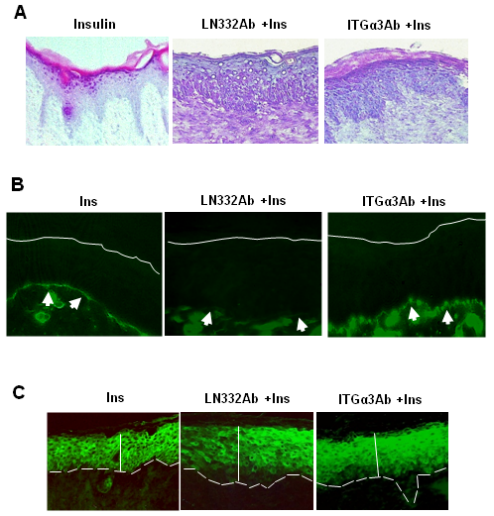
**LN332 and Integrin α3 inhibition decrease matrix and keratin 10 production**. Wound tissues were collected at wound closure and sections made. **(A) **Representative hematoxylin and eosin-stained sections show that in the LN332 and integrin α3 antibody-treated mice the epidermis is morphologically less differentiated, the basal keratinocytes are not well aligned, the reticular ridges not well formed, and the number of layers of keratinocytes is higher. **(B) **Immunolabeling for Collagen IV, a marker for basement membrane development, shows that the LN332 antibody causes a dramatic decrease in basement membrane deposition and integrin α3 antibody results in a basement membrane that contains many densities that contributes to irregularity of Collagen IV deposition. Solid lines demark the apical surface of the epidermis and arrowheads point to basement membrane). **(C) **Immunolabeling for keratin 10, a marker for keratinocyte differentiation, shows that the basal layers of the epidermis are disorganized and undifferentiated when LN332 and integrin α3 antibodies are applied to the wounds. Dashed lines delineate the basal surface of the epidermis and the vertical lines delineate the thickness of the epidermis.

These results suggest that *in vivo *both α3 and LN332 are involved in insulin-induced acceleration of wound closure. To test this possibility, we applied the function-blocking Abs against both proteins to mouse excision wounds, and found that inhibition of LN332 (Fig. 7A, B) or integrin α3 (Fig. 7C, D) resulted in delays in healing, primarily at early times after wounding. Histological examination shortly after wound closure showed that both inhibition of LN332 or integrin α3 resulted in a less mature epidermis (Fig. 8A). When the antibodies to these two molecules were applied, no reticular ridges were seen, the basal cells were not well defined, the interactions of the epidermis with the dermis were less well defined, and appendages were not seen. Furthermore, staining for Collagen IV (Fig. 8B), a component of basement membrane, showed that in the wounds treated with the function-inhibiting Abs for LN332, the basement membrane was not well developed, and when treated with α3 integrin, the basement membrane was irregular containing many dense areas of Collagen IV deposition (compare with the staining for wounds treated with insulin alone). When we stained for keratin 10 (Fig. 8C), a marker of keratinocyte differentiation [25], the basal keratinocytes of the wounds treated with the inhibiting antibodies did not express keratin 10. These results suggest that insulin promotes epithelial basement membrane deposition and keratinocyte differentiation.

## Discussion

Previous work has shown that insulin improves wound healing, but the mechanisms of its action on healing have not been delineated. We used a variety of cell and molecular approaches to determine the effects of insulin on cutaneous wounds and have shown that insulin: (1) stimulates keratinocyte migration in a dose- and time-dependent manner; (2) acts in an insulin-receptor-dependent but EGF/EGF-R-independent manner; (3) stimulates keratinocyte migration through the PI3K-Akt-Rac1 pathway; (4) stimulates keratinocytes to produce integrin α3 and LN332, and cell migration *in vitro *and *in vivo *is dependent on these molecules. The ability of this hormone to stimulate a variety of cell functions important for wound healing in an insulin-receptor-dependent manner creates the ability to target processes that are dependent only on insulin. Conversely, the ability to stimulate both the insulin and IGF-1 receptors may broaden the applicability of insulin in different wound conditions, particularly when one receptor may be missing or dysfunctional (e.g. type II diabetes).

Understanding the processes by which insulin accelerates wound closure is important because it will provide insight into potential manipulation of the healing process using this hormone as well as the signaling molecules it activates. The results presented here show that stimulation of keratinocyte migration by insulin involves the PI3K-Akt pathway, and identifies Rac1, a small GTPase, as a molecule activated downstream of PI3K-Akt. Support for these conclusions includes the fact that these molecules are activated upon insulin stimulation, and inhibition of each molecule with specific inhibitors or dominant negative proteins prevents insulin-induced keratinocyte migration.

Rac1 is known to regulate actin assembly [26] and to stimulate formation of lamellipodia [27], thereby promoting cell movement in response to external signals from cytokines, growth factors and/or the ECM. Our results support insulin-induced Rac1 activation in keratinocytes, as shown by Rac1 translocation from the cytosol to the cell membrane, the formation of membrane ruffles upon insulin stimulation, and the increased levels of active Rac1 identified by the pull down assay. Moreover, we show that Rac1 activation is dependent on the PI3K-Akt signaling pathway in keratinocyte migration. Two recent papers showed a strong activation of the PI3K pathway in the wound margin keratinocytes [28,29]. Furthermore, epidermal growth factor (EGF) or heregulin (HRG) have been found to stimulate keratinocyte migration through PI3K/Akt signaling [30]. These results, coupled with our findings presented here, show that insulin can replace some well known growth factors in wound healing.

The effects of insulin on keratinocyte migration described above, led us to hypothesize that insulin-accelerated wound healing involves increased expression of the integrin α3β1 in keratinocytes as well as an increase in the levels of LN332. The latter protein is a matrix molecule secreted by migrating keratinocytes at the leading edge [31], where it mediates keratinocyte polarity and persistent migration [32,33]. After wounding, quiescent epidermal keratinocytes are activated and express the integrins α6β4 and α3β1, which mediate their migration on LN332 and facilitate the development of the BM [34]. Our results show that insulin stimulates keratinocyte integrin α3 expression and LN332 deposition, and that inhibition of these proteins *in vitro *or *in vivo *inhibits insulin-induced keratinocyte migration and wound healing, strongly suggesting an important role for these molecules in insulin stimulation of healing. This is a particularly important finding, as the relationship between LN332 and migration has been controversial. Indeed, previous studies have implicated LN332 in promoting and inhibiting cell migration [34-36]; our results support a role for LN332 in promoting keratinocyte migration.

In addition to its effects on keratinocyte migration during wound healing, insulin also promotes attachment of the epidermis to the dermis, the appearance of a well-organized epidermis, increased numbers of skin appendages, and more dermal papilla and epidermal reticular ridges. We also found that inhibiting integrin α3 and LN332 resulted in lack of full epidermal differentiation (as shown by disorganization of the epidermis) and decreased formation of dermal papilla and epidermal reticular ridges.

Our results suggest that the direct application of insulin to chronic wounds may improve wound healing by compensating for a deficiency of insulin and/or IGF-1 in the injured area. Previous studies have shown that the IGF-1 level is decreased in both streptozotocin (STZ)-induced diabetic and normal rat incision wounds [37], and we have also detected a decrease in insulin levels in rat scald wounds (unpublished data). This insulin and IGF-1 deficiency in the wound, along with our data showing that insulin can function through stimulation of both insulin and IGF-1 receptors, suggests that direct application of insulin to the wound area may improve healing through activation of both receptors. Indeed, it has been shown that topical application of insulin accelerates healing of infected cutaneous ulcerations in diabetic mice [12], showing that insulin is promising for treatment of these types of wounds. However, it has also been shown that leptin-deficient obese/obese (ob/ob) diabetic mice have dysfunctional signaling during wound healing [38], suggesting that in these mice mediating the effect of insulin on wounds might be different from those in normal wounds or wounds in diabetic people.

When compared with other growth factors used to promote wound repair, insulin treatment is likely to be much less expensive, more readily available, and has already been approved by the FDA for human use. Moreover, a significant numbers of reports have described that treatment with growth factors, including most of the growth factors that have been used clinically, or increased expression of growth factor receptors, lead to carcinogenesis [39-41]. In contrast, insulin is safe, as shown by its use for nearly a hundred years, hence it is likely safer than growth factor alternatives. When choosing a concentration of insulin for possible wound therapy, it is important to remember that, although the highest concentrations of insulin resulted in the greatest keratinocyte migration, such doses may alter blood glucose levels *in vivo*.

## Conclusion

In conclusion, we have shown that insulin interacts with its receptor and affects multiple aspects of keratinocyte behavior, including stimulation of cell motility, increased expression of the cell surface adhesion molecule integrin α3 and enhanced secretion of the ECM molecule LN332. Furthermore, these effects extend to the dermis where we observe a higher degree of tissue restoration than seen in the control. These results strongly suggest that insulin improves wound healing through an integrated effect not only on re-epithelialization but also on the underlying granulation tissue. Therefore, insulin treatment may prove to be a powerful therapy for the treatment of impaired wounds, especially given the fact that insulin has been used in humans for a century without serious adverse consequences.

## Methods

### Reagents

The human keratinocyte cell line HaCaT was a gift from DKFZ (German cancer research center). DMEM was purchased from Mediatech and FBS from Atlanta Biologicals. Anti-RhoA (sc-179) Abs was purchased from Santa Cruz Biotechnology. Anti-phospho-Akt Ser (473), anti-insulin receptor (29B4) Abs and the PI-3K inhibitors, LY294002 came from Cell Signaling. The TRITC-anti-mouse Rac1 Ab was purchased from Becton Dickinson and Company. And the anti-human LN332 (P3H9-2) and anti-human integrin α3 (P1B5) Abs were purchased from Chemicon. The anti-mouse cytokeratin 10 Ab and rabbit polyclonal to type collagen IV Ab were from Abcam. Anti-GAPDH Ab was obtained from RDI Research Diagnostics. Recombinant human insulin and mitomycin C were purchased from Sigma. Humulin Ultralene human insulin (rDNA origin) extended zinc suspension was purchased from Eli Lilly and Company. EGF-R inhibitor AG1478 and IGF-1 receptor tyrosine kinase inhibitor, Picropodophyllin were purchased from Calbiochem. FITC-conjugated goat anti-mouse immunoglobulin came from DAKO, and anti-mouse Texas Red Ab from Amersham.

### In vivo wound model

C57BL/6J mice were purchased from The Jackson Laboratory (USA), and housed at the University of California, Riverside (UCR) vivarium. All experimental protocols were approved by the UCR Institutional Animal Care and Use Committee. Experiments were performed using 8–12 wk old mice. Mice were anesthetized with a single intraperitoneal injection of ketamine (80 mg/kg body weight)/xylazine (16 mg/kg body weight). Full-thickness 7 mm punch wounds (excision of the skin and the underlying panniculus carnosus) were made on the back of the mice. The wounds were then treated as indicated for the various experiments and covered with a transparent dressing (Bioclusive, Johnson & Johnson Medical Limited, USA) until day 3 after wounding. After this time, the dressings were removed, and the wounds were exposed. Healing was monitored by taking photographs at the indicated time points. In addition, the wound area was drawn on a transparent plastic film for further comparisons of wound area at each time point. For graphing purposes, wound closure was expressed as percentage of wound area (area of wound measured at any one day post wounding divided by the initial wound area).

### Histology

At day 3 after wounding, as well as the day that complete healing occurred, animals were anesthetized, and skin samples consisting of the wounded area plus 5 mm of the surrounding unwounded and/or healed skin area were collected. Tissues were fixed in 4% pareformaldehyde for 2 h and incubated in 0.1 M glycine/PBS for 1 h followed by 15% and 30% sucrose before embedding in OCT (Tissue-Tek, Sakura Finetek. USA, Inc), freezing in a slush of ethanol/dry ice, and stored in -80 C. After sample collection, the mice were euthanized using CO_2_. The extent of migration of the keratinocyte was measured in sections stained with Hematoxylin and Eosin. The length of migration tongue was determined by the distance from the margin of the wound to the tip of the migrating keratinocytes.

### Cell Culture

The human keratinocyte cell line HaCaT was cultured in 5% CO_2 _at 37°C in DMEM supplemented with 10% FBS, 10 units/ml penicillin, and 10 μg/ml streptomycin sulfate (GIBCO, Invitrogen Corporation).

### Immunoblotting

Cells were treated as indicated, and then washed with ice-cold 1 × PBS, and lysed on ice with lysis buffer containing 0.5% Triton X100, 0.5% Nonidet P-40, 10 mM Tris, pH 7.5, 2.5 mM KCl, 150 mM NaCl, 30 mM b-glycerophosphate, 50 mM NaF, 1 mM Na3VO4, 0.1% SDS and additional protease and phosphatase inhibitor cocktails (Sigma). Protein concentrations were measured using the DC protein assay kit (Bio-Rad). Equal amounts of protein in the cell extracts were mixed with sample buffer, boiled, and analyzed using 10% acrylamide SDS-PAGE. Immunoblotting was performed with the indicated primary Abs and the appropriate HRP-conjugated secondary Abs, followed by incubation with West Dura extended duration substrate (Pierce Biotechnology). Blots were then re-probed for house keeping proteins to show equal loading. For LN332 and integrin α3, protein extracts were prepared as indicated above, but were mixed with non-reducing sample buffer, and, for LN332 detection, samples were boiled. The extracts were then analyzed using 7.5% acrylamide SDS-PAGE, followed by immunoblotting as described above.

### In vitro Migration Assays

We used two types of assays: (i) The cloning ring assay in which 2.0 × 10^4 ^HaCaT cells were plated in a cloning ring 6 mm diameter set within a 35-mm cell culture dish. Four hours after seeding, the cylinder was removed, the cell edges were marked, and migration was measured at the indicated times by measuring the distance from the initial cell edge to the edge of the migrating cells; (ii) For the scratch assay the HaCaT cells were plated in cell culture dishes and after reaching confluence, scratch wounds were made as previously described [42]. Briefly, we used the small end of a 1 ml pipet tip to scratch the cells on a 35 mm plate, and then marked the edges of the scratches. Cell migration was measured at the indicated times by measuring the distance from the initial cell edge to the edge of the migrating cells. The scratch assay was used when we performed transfection experiments to avoid trypsinization and re-plating into the cloning ring.

### Transient Adenoviral Transfection

Recombinant adenovirus containing the constitutively active mutant of Akt (Akt-CA), the dominant-negative mutant of Akt (Akt-DN), and the parental adenoviral vector (referred to as null) were gifts from John Shyy (UCR, Biomedical Sciences Division). Cells were infected with various recombinant adenoviruses at 50 moi; media was changed after 5 h of incubation with adenovirus. After 48 h of infection, cells were subjected to further treatment, as indicated in the Results section. Plasmids expressing the constitutively active form of Rac1 (V12, Rac1-CA), the dominant-negative mutant Rac1 (N17, Rac1-DN) and the wild type Rac1 (Rac1-WT; gifts from Dr. Miguel Del Pozo, Centro National de Investigaciones Cardiovasculares, Madrid, Spain) were transfected using Lipofectin (Invitrogen, USA) according to the manufacturer's protocol. Briefly, one day before transfection, the cells were plated in growth media without antibiotics. Transfections were performed at 40–60% cell confluence. 2 μg of DNA in 100 μl of DMEM without serum, were mixed gently with 5 μl Lipofectin in 100 μl of MDEM without serum. This mixture was incubated for 15 min at room temperature and then added to the cells which were then incubated at 37°C and 5% CO_2 _for 16 h. At this time the medium was replaced with medium containing serum and analyzed at the appropriate times.

### Immunolabeling

Cells were cultured in chamber slides (Nunc), and fixed in 4% paraformaldehyde for 20 min, rinsed with PBS, incubated in PBS containing 0.1 M glycine for 20 min, and blocked with 3% BSA, 0.1%Triton X-100 in PBS for 30 min. Primary Abs in 1% BSA/PBS were applied to the sample for 2 h at room temperature, washed, and incubated with 1:50 dilution of FITC or Texas red-conjugated secondary Abs for 1 h at room temperature. After washing, the cells were mounted in Vectashield containing DAPI (Vector Laboratories, Inc. Berlingame, CA). Immunofluorescence was visualized and imaged using a Leica SP2 laser scanning confocal microscope. For frozen tissues, 8-μm cryosections were washed in 1 × PBS to remove the OCT, fixed in 2% paraformaldehyde for 10 min, incubated in 0.1 M glycine in 1 × PBS, followed by the primary and secondary Abs using the same procedure as indicated above.

### Statistical Analysis

Data are shown as mean × SD. Data analysis was performed using the unpaired Student's t-test on raw data using GraphPad Instat software (GraphPad Software Inc.). Statistical comparison between more than two groups was performed by One-way ANOVA.

## Abbreviations

BM: basement membrane; CA: constitutively active; DN: dominant negative; WT: wild type; LN332: laminin 332

## Authors' contributions

YL conducted the majority of the experiments and drafted the manuscript. MLP participated in data acquisition and manuscript preparation. MY assisted in experimental design. MMG conceived of the study, participated in its design and coordination, and helped in the writing of the manuscript.
